# Interferon-β1b Increases Th2 Response in Neuromyelitis Optica

**DOI:** 10.3390/ijms131012213

**Published:** 2012-09-25

**Authors:** Hideto Nakajima, Takafumi Hosokawa, Yoshimitu Doi, Toshiyuki Ikemoto, Shimon Ishida, Fumiharu Kimura, Toshiaki Hanafusa

**Affiliations:** 1 Division of Neurology, Department of Internal Medicine I, Osaka Medical College, Takatsuki, Osaka 569-8686, Japan; E-Mails: in1237@poh.osaka-med.ac.jp (T.H.); in1311@poh.osaka-med.ac.jp (Y.D.); in1212@poh.osaka-med.ac.jp (S.I.); in1110@poh.osaka-med.ac.jp (F.K.); hanafusa@poh.osaka-med.ac.jp (T.H.); 2 Department of Central Laboratory, Osaka Medical College, Takatsuki, Osaka 569-8686, Japan; E-Mail: kns008@poh.osaka-med.ac.jp

**Keywords:** neuromyelitis optica, multiple sclerosis, IFN-â1b, chemokine receptor, CCR5, CXCR3, CCR4, Th1, Th2

## Abstract

A Japanese randomized controlled study showed that Interferon â (IFN-â1b) therapy is clinically effective in decreasing the frequency of attacks in multiple sclerosis (MS), even in optico-spinal MS (OSMS). However, recent studies have shown that IFN-â (IFN-â1a/IFN-â1b) treatment was not effective in neuromyelitis optica (NMO) patients and that the diminished benefit of IFN-â treatment in NMO may be due to different immune responses to IFN-â. We determined longitudinally the expression of CCR5, CXCR3 and CCR4 on CD4+ T and CD8+ T cells in the blood from patients with NMO and MS treated with IFN-â1b. During a 12-month period of IFN-â1b therapy, the annualized relapse rate decreased in MS patients but not in NMO patients. There was no significant difference in the expression of the chemokine receptors between NMO and MS at baseline. The percentages of CD4+CCR5+ and CD4+CXCR3+ T cells, representative of the Th1 response, were decreased in both NMO and MS after treatment. The percentage of CD4+CCR4+ T cells, representative of the Th2 response, was decreased in MS, but those for NMO was significantly increased compared with the pretreatment levels. Our results indicate that IFN-â1b-induced up-modulation of the Th2 response in NMO patients may be the source of differences in the therapeutic response to IFN-â1b therapy. In the present study, Th2 predominance is involved in the pathogenesis of NMO.

## 1. Introduction

Interferon â (IFN-â) has been used for years in the treatment of relapsing-remitting multiple sclerosis (RRMS) with proven clinical efficacy. The therapeutic benefit of IFN-â (IFN-â1a/ IFN-â1b) appears to be based on immunomodulatory mechanisms. Previous data has shown divergent evidence regarding the effect of IFN-â on the Th1-Th2 balance in patients with MS treated with IFN-â [1]. Overall, most studies indicate a suppression of the generation of Th1 cytokines following treatment with IFN-â. Decreased levels of Th1 cytokines, such as IL-12, IFN-ã, and tumor necrosis factor á, have been described. Although various T cell subsets produce IL-10 under receptor stimulation, IL-10 evidently acts as an anti-inflammatory or Th2 cytokine. Elevated levels of IL-10 in the mononuclear cell fraction and in the serum and cerebrospinal fluid (CSF) of patients with MS treated with IFN-â have been reported. However, other reports indicate a more general suppression of both Th1 and Th2 subsets in patients treated with IFN-â [2,3]. Although the literature remains contradictory regarding the effect of IFN-â on the Th1-Th2 balance, IFN-â might inhibit Th1 cytokines and stimulate Th2 cytokines [4,5].

Neuromyelitis optica (NMO) is an idiopathic inflammatory disease of the central nervous system (CNS) that mainly affects the optic nerve and spinal cord [6]. Although the clinical characteristics of this disease are similar to those of MS, recent clinical, histopathological, and serological findings strongly suggest the involvement of the humoral immune system in NMO [7–9] and that the detection of serum anti-aquaporin-4 (AQP4) antibody can be used to distinguish NMO from MS [10,11].

In Japan, the efficacy of IFN-â1b for MS was also reported in a randomized controlled trial and IFN-â1b is the first-line drug that is now publicly approved for MS. This Japanese randomized controlled study showed that IFN-â1b therapy is effective in decreasing attack frequency even in optico-spinal MS (OSMS) [12]. However, recent studies have shown that IFN-â treatment was not effective in NMO/OSMS patients [13,14]. Since humoral-mediated immunity plays an important role in the physiopathology of NMO, the diminished benefit of IFN-â therapy in NMO may be induced by a shift in the immunological profile toward a Th2-dominant system in response to IFN-â. Chemokines are low-molecular-weight cytokines produced in inflamed tissue, mediating the recruitment of specific leukocyte populations expressing chemokine receptors. They also play an important role in immune-regulatory activation such as cytokine production and Th1/Th2 cell induction. CXCR3 and CCR5 are expressed on Th1 cells, and CCR3 and CCR4 are expressed on Th2 cells. Recent studies have shown that T cells expressing CCR5 and CXCR3 can be detected within the perivascular lesions of brains from patients with MS [15–17]. Increased expression of CXCR3 on CD4+ T cells in peripheral blood from MS patients compared to those of healthy individuals has also been reported [18,19]. A few studies have reported the expression of chemokine receptors on peripheral blood in NMO patients, but there have been no studies concerning the effect of IFN-â therapy on Th1-/Th2- related chemokine receptor expression in NMO patients [19]. Thus, the aim of this study was to determine the expression of CCR5, CXCR3, and CCR4 on blood CD4+ T and CD8+ T cells from patients with NMO and MS treated with IFN-â1b (Betaferon). The expression of the chemokine receptors was measured longitudinally in these patients over a 12-month period of treatment.

## 2. Results

### 2.1. Comparison of Th1/Th2-Related Chemokine Receptor Expression at Baseline

The percentage of CD4+ or CD8+ T cells expressing the Th1- and Th2-specific chemokine receptors among CD3+ T cells was determined by three-color flow cytometry using anti-CD3 antibody and anti-CD4 antibody or anti-CD8 antibody and one of the following anti-chemokine receptor antibodies: anti-CXCR3 antibody, anti-CCR5 antibody, and anti-CCR4 antibody. CXCR3 and CCR5 are chemokine receptors expressed on Th1 cells.

As shown in Table 1, the percentage of CD4+CXCR3+ cells for the NMO group was significantly higher than that for the NMO group and the healthy control (HC) group at baseline. The CCR5 expressions on CD4+ or CD8+ T cells for the NMO group tended to be lower than those for the MS, but differences did not reach statistical significance (Table 1). Thus, the percentage of CXCR3+ Th1 indicates the level of NMO activity. On the other hand, the percentage of CCR5+ Th1 may reflect MS activity rather than NMO activity. As for the expression of CCR4 on Th2 cells, the CCR4 expressions on CD4+ or CD8+ T cells for the NMO tended to be higher than those for MS, but differences did not reach statistical significance (Table 1).

### 2.2. Changes in Chemokine Receptor Expression by IFN-â1b Treatment

Next, the expression of the chemokine receptors was measured longitudinally in 5 NMO and 6 MS patients treated with IFN-â1b (Betaferon) for 12 months. The MS group was younger, but there were no significant differences in disease duration, EDSS scores, or annualized relapse rate before IFN-â1b treatment (Table 2). The annualized relapse rate in the MS group decreased after IFN-â1b treatment, but not in the NMO group (Table 2).

**Figure 1:**
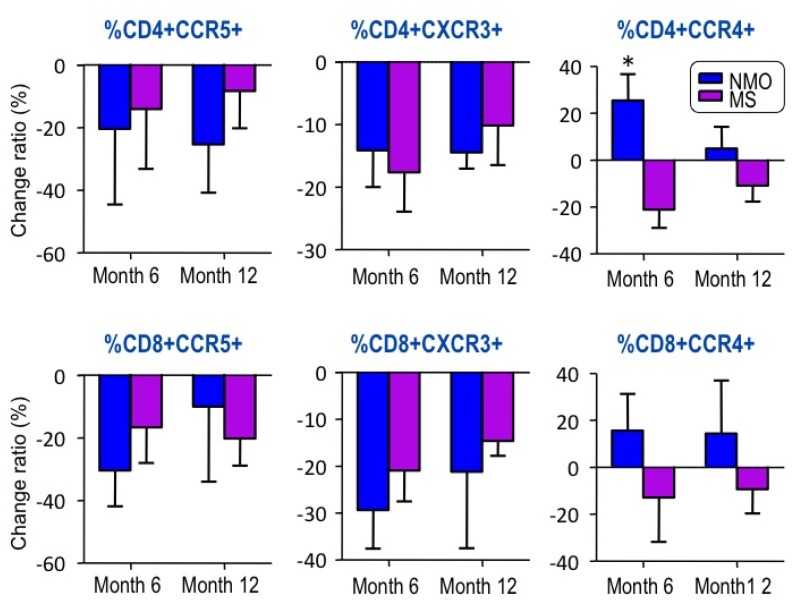
Changes in expression of CCR4, CCR5 and CXCR3 on CD4+ and CD8+ T cells between samples obtained from patients with NMO (blue) and MS (purple) at baseline and after 6 or 12 months of IFN-â1b treatment.

We analyzed changes in expression of CCR4, CCR5 and CXCR3 on CD4+ and CD8+ T cells between samples obtained at baseline and after 6 months or 12 months of IFN-â1b treatment. After IFN-â1b treatment, the percentages of CD4+CCR5+ cells and CD4+CXCR3+ cells, representative of Th1 response, were decreased in both groups (Figure 1). The percentage of CD4+CCR4+, representative of Th2 response, was decreased in MS, but significantly increased in NMO compared with the pretreatment levels (Figure 1). Our results indicate that IFN-â1b-induced up-modulation of the Th2 response in NMO may be the source of the differences between MS and NMO in the therapeutic response to IFN-â1b. Th2 predominance may be involved in the pathogenesis of NMO.

## 3. Discussion

Recent reports have suggested that IFN-â has no effect on or exacerbates NMO [13,14,20–22]. In the present study, the annualized relapse rate in the MS group was significantly decreased one year after IFN-â1b treatment while that in the NMO group showed no significant decrease. After IFN-â1b treatment, the percentage of cells that were CD4+CCR4+, representative of a Th2 response, decreased in the MS group, but was significantly increased in the NMO group compared with pretreatment levels (Figure 1). An up-modulation of Th2 response caused by IFN-â1b treatment appears to be characteristic for patients with NMO. As the present study examined only a few cases, there could have been a bias in the selection of patients for IFN-â1b treatment. However, it was considered of value for demonstrating the immunological mechanism of IFN-â1b on the Th2 response in the pathogenesis of NMO.

In the present study, we assessed the expression of CXCR3 and CCR5 (Th1 cell markers) and CCR4 (Th2 cell marker) on the peripheral T cells of MS and NMO patients to ascertain the relationship with disease activity. The results showed that the percentage of CD4+ CXCR3+ cells was higher for the MS group compared to the HC group, thus suggesting that the percentage of Th1 cells is higher in MS (Table 1). CCR5 is another Th1 cell marker, but there was no clear difference in the percentage of CCR5+ cells between the MS group and HC. Although the percentage of CD4+CCR4+ T cells was not different between MS and HC, Th1 CXCR3 cells may indicate the level of MS activity. The present investigation showed that the percentage of CD4+CXCR3+ cells was significantly higher in NMO patients than in HC, and that values were more remarkable than in the MS group. The anti-AQP4 antibody has been identified as a disease-specific autoantibody in NMO patients, and the pathogenesis of NMO is dominated by humoral mechanisms [23,24]. Our results do not support a distinct Th2 dominancy in NMO and are in accord with the results of previous reports [19]. However, NMO patients have a tendency to demonstrate decreased CCR5 and the increased CCR4 expression compared to MS patients.

IFN-â is widely used as first-line treatment for MS [25]. To date, the therapeutic effect of IFN-â in MS has mainly been attributed to direct anti-inflammatory effects, either systemically through alteration of immune cell states or locally at the BBB through down-regulation of several molecules, and previous reports have demonstrated reduced CXCR3+ Th1 activities or IL-10 (Th2 cytokine) induction by IFN-â treatment in MS patients [26]. However, a recent study demonstrated induction of IP-10/CXCL10 mRNA in PBMC derived from MS patients treated with IFN-â in addition to other pro-inflammatory molecules, concluding a more complex mechanism exists [27]. IFN-â is ineffective or even harmful in cases of NMO. Krumbholz *et al*. described that IFN-â therapy induces a potent B-cell survival factor, B-cell activating factor of the TNF family (BAFF) [28]. The systemic induction of BAFF by IFN-â therapy may facilitate the production of various autoantibodies and of IFN-neutralizing antibodies. Elevated systemic levels of BAFF have been observed in serum of patients with systemic, B-cell-related autoimmune diseases such as Sjögren’s syndrome [29], systemic lupus erythematosus (SLE) [30] and Wegener’s granulomatosis [31]. Therefore, NMO patients who have major B-cell involvement may benefit less from IFN-â therapy. A recent report indicated an increase of the anti-AQP4 antibody titer associated with treatment by IFN-â in an NMO patient [32]. In terms of the impact of IFN-â on Th2 cells, Karni *et al*. showed that IFN-â did not lead to increased expression of CCR4+ Th2 cells in MS patients [33]. Our results also showed no significant changes in CCR4+ expression in the MS group. However, our study demonstrated that after IFN-â treatment, the percentage of CD4+CCR4+ significantly increased in the NMO group, suggesting up-modulation of the Th2 response by IFN-â in autoimmune diseases involving the humoral immune system. Recent studies reported that Th17 cells express CCR4 and CCR6 [34,35]. We did not examine expression of CCR6 and the Th17 subset. Recent animal and human studies have shown that Th17-related cytokines and chemokines play a key role in the pathogenesis of NMO. Axtell *et al*. demonstrated that IFN-â treatment effectively blocked disease symptoms in EAE mice induced with Th1 cells, but exacerbated disease symptoms in Th17-induced EAE mice [36]. In addition, levels of IL-17 were elevated in the CSF of patients with NMO compared to MS [37]. Although the effect of IFN-â treatment on Th17- and Th2-related responses in NMO pathogenesis remains unclear, our findings suggest that Th2 and Th17 lineages may be involved in the pathogenesis of NMO.

## 4. Experimental Section

### 4.1. Subjects

Ten patients with NMO (all women; mean ± SD age, 45.1 ± 12.6 years) diagnosed by the Wingerchuk criteria [38] and 16 patients with MS (five men and 11 women; mean ± SD age, 35.9 ± 12.9 years) diagnosed by the McDonald criteria [39] were enrolled in this study at the Department of Neurology, Osaka Medical College Hospital, located in Takatsuki, Japan (Table 3). Anti-AQP4 antibody assays were performed in 21 patients with spinal cord lesions, but were not performed in four patients without a spinal cord lesion who fulfilled the McDonald criteria but did not fulfill the Wingerchuk criteria regardless of the results of the assay. In addition, 14 healthy control subjects (four men and six women; mean ± SD age, 33.4 ± 7.2 years) were included as the HC group. Medical records of the patients including disease severity and spinal magnetic resonance imaging (MRI) findings were also analyzed retrospectively. Disease severity was scored in all NMO and MS patients using Kurtzke’s Expanded Disability Status Scale (EDSS) [40]. The expression of CCR5, CXCR3, and CCR4 on CD4+ T or CD8+ T cells in the blood was measured by three-color flow cytometry before IFN-â1b or immunosuppressive therapies.

The demographic features of patients are summarized in Table 3. The number of relapses, EDSS score, and frequency of longitudinally extensive spinal cord lesions (LESCL) were significantly higher in NMO patients than in MS patients, while the female preponderance, age at onset, and disease duration did not differ significantly between NMO and MS patients.

Of these cases, expression of the chemokine receptors was measured longitudinally in five NMO and six MS patients treated with IFN-â1b (Betaferon) over the course of 12 months. These patients received eight MIU IFN-â1b subcutaneously every other day. Before the initiation of IFN-â1b therapy, the five NMO patients had been diagnosed with clinically definite MS, classified as OSMS, in which clinically determined significant lesions were confined to the optic nerve and spinal cord. In these patients, anti-AQP4 antibody was measured during the disease course after initiation of IFN-â1b therapy. As all these patients were positive for Anti-AQP4 antibody, they were retrospectively diagnosed with definite NMO. Informed consent was obtained from all participants according to the Declaration of Helsinki.

### 4.2. Flow Cytometry

Chemokine receptor expression was assessed as described previously [18,41,42]. Venous blood was collected in heparinized tubes and analyzed within two hours after sampling. Whole blood samples were labeled with directly conjugated monoclonal antibodies, according to the instructions of the manufacturer, using anti-CD3 PerCP (Becton Dickinson, San Jose, CA, USA), anti-CD4 FITC, anti-CD8 FITC (Pharmingen, San Diego, CA, USA), anti-CCR4 PE, anti-CXCR3 PE (Pharmingen, San Diego, CA, USA) and anti-CCR2 PE (Dako, Kyoto, Japan), in addition to isotype-specific antibody controls. Cells were fixed in 2% paraformaldehyde and stored in the dark before analysis using a FACS flow cytometer (Becton Dickinson). Flow cytometry data was processed using CellQuest software (Becton Dickinson). Data are reported as percentages of all T cells (identified as CD3+ cells) staining positively for CD4+CXCR3+, CD8+CXCR3+, CD4+CCR4+, or CD8+CCR4+.

### 4.3. Statistical Analysis

Descriptive data was compared using Fisher’s exact test and Mann-Whitney U test for proportion, and Wilcoxon’s signed rank test for paired continuous measures. Comparisons between expressions of chemokine receptors in blood from NMO patients, MS patients, and healthy controls were performed by Mann–Whitney U test and Kruskall-Wallis tests. Values of *p* < 0.05 were considered statistically significant.

## 5. Conclusions

During the 12-month period of IFN-â1b therapy, the annualized relapse rate of the MS group decreased, but that of the NMO group did not. The percentages of CD4+CCR5+ and CD4+CXCR3+ T cells, representative of Th1 response, were decreased in both NMO and MS. The percentages of CD4+CCR4+ T cells, representative of Th2 response, were decreased in MS but significantly increased in NMO compared with pretreatment levels. Our results indicate that IFN-â1b-induced up-modulation of Th2 responses in NMO may be the source of the difference in the therapeutic responses of MS and NMO to IFN-â1b therapy. Also, Th2 predominance may be involved in the pathogenesis of NMO.

## Figures and Tables

**Table 1:** Baseline of chemokine receptor expression on blood T cells.

	NMO (*n* = 10)	MS (*n* = 16)	HC (*n* = 14)
CD4+CCR5+	4.71 ± 1.44	6.17 ± 2.74	5.97 ± 0.99
CD8+CCR5+	9.53 ± 3.69	16.14 ± 8.33	15.40 ± 4.02 *
CD4+CXCR3+	24.58 ± 3.44	18.81 ± 5.05 **	15.75 ± 2.52 ***
CD8+CXCR3+	24.55 ± 8.74	26.58 ± 9.52	23.0 ± 3.66
CD4+CCR4+	13.48 ± 5.15	10.94 ± 3.24	11.34 ± 1.95
CD8+CCR4+	2.44 ± 2.43	1.06 ± 0.65	0.71 ± 0.37

Values are expressed as means ± SDs. NMO, neuromyelitis optica; MS, multiple sclerosis; HC, healthy control. Data are reported as percentages of all T cells staining positively for CD4+CXCR3+, CD8+CXCR3+, CD4+CCR4+, or CD8+CCR4+.

* *p* < 0.05 compared to NMO.

** *p* < 0.05 compared to NMO.

*** *p* < 0.005 compared to NMO.

**Table 2:** Demographic features of neuromyelitis optica (NMO) and multiple sclerosis (MS) patients treated with Interferon â (IFN-â1b) longitudinaly.

	NMO (*n* = 5)		MS (*n* = 6)		*p* Value
Age	55.3		42.2		<0.05
Disease duration (years)	9.1		6.8		ns
EDSS score	5.1		3.3		ns
Annualized relapse rate before IFN-â1b	2	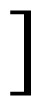 ns	2.3	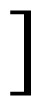 <0.05	ns
Annualized relapse rate after start of IFN-â1b	2		0.3		ns

NMO, neuromyelitis optica; MS, multiple sclerosis; EDSS, expanded disability status scale.

**Table 3:** Patient demographics.

	NMO (*n* = 10)	MS (*n* = 16)	HC (*n* = 10)	*p* Value (NMO *vs.* MS)
Sex (M:F)	0:10	5:11	4:6	ns
Age	45.1 ± 12.6	35.9 ± 12.9	33.4 ± 7.2	ns
Age at onset (years)	37.2 ± 11.5	32.9 ± 12.8		ns
Disease duration (years)	7.5 ± 8.8	3.5 ± 4.8		ns
Number of relapse	5.6 ± 4.5	2.4 ± 1.8		<0.05
EDSS score	6.5 ± 2.1	2.9 ± 0.8		<0.0001
Treatment	5: IFN-â1b 5: treated with PSL and/or AZT	12: IFN-â1b		
Anti-AQP4 antibody	100%	0%		<0.0001
LESCL	80%	0%		<0.0001

Values are expressed as means ± SDs. NMO, neuromyelitis optica; MS, multiple sclerosis; HC, healthy control; EDSS, expanded disability status scale; PSL, prednisolone; AZT, azathioprine; AQP4, aquaporin-4; LESCL, longitudinally extensive spinal cord lesions.
